# Higher Status Honesty Is Worth More: The Effect of Social Status on Honesty Evaluation

**DOI:** 10.3389/fpsyg.2018.00350

**Published:** 2018-03-20

**Authors:** Philip R. Blue, Jie Hu, Xiaolin Zhou

**Affiliations:** ^1^Center for Brain and Cognitive Sciences and School of Psychological and Cognitive Sciences, Peking University, Beijing, China; ^2^Key Laboratory of Machine Perception, Ministry of Education, Peking University, Beijing, China; ^3^Beijing Key Laboratory of Behavior and Mental Health, Peking University, Beijing, China; ^4^PKU-IDG/McGovern Institute for Brain Research, Peking University, Beijing, China

**Keywords:** social status, promise, trust, trust game, social hierarchy, ERP, MFN, P300

## Abstract

Promises are crucial for maintaining trust in social hierarchies. It is well known that not all promises are kept; yet the effect of social status on responses to promises being kept or broken is far from understood, as are the neural processes underlying this effect. Here we manipulated participants’ social status before measuring their investment behavior as Investor in iterated Trust Game (TG). Participants decided how much to invest in their partners, who acted as Trustees in TG, after being informed that their partners of higher or lower social status either promised to return half of the multiplied sum (4 × invested amount), did not promise, or had no opportunity to promise. Event-related potentials (ERPs) were recorded when the participants saw the Trustees’ decisions in which the partners always returned half of the time, regardless of the experimental conditions. Trustee decisions to return or not after promising to do so were defined as honesty and dishonesty, respectively. Behaviorally, participants invested more when Trustees promised than when Trustees had no opportunity to promise, and this effect was greater for higher status than lower status Trustees. Neurally, when viewing Trustees’ return decisions, participants’ medial frontal negativity (MFN) responses (250–310 ms post onset) were more negative when Trustees did not return than when they did return, suggesting that not returning was an expectancy violation. P300 responses were only sensitive to higher status return feedback, and were more positive-going for higher status partner returns than for lower status partner returns, suggesting that higher status returns may have been more rewarding/motivationally significant. Importantly, only participants in low subjective socioeconomic status (SES) evidenced an increased P300 effect for higher status than lower status honesty (honesty – dishonesty), suggesting that higher status honesty was especially rewarding/motivationally significant for participants with low SES. Taken together, our results suggest that in an earlier time window, MFN encodes return valence, regardless of honesty or social status, which are addressed in a later cognitive appraisal process (P300). Our findings suggest that social status influences honesty perception at both a behavioral and neural level, and that subjective SES may modulate this effect.

## Introduction

Promises are crucial for creating trust in situations where trust does not yet exist (Malhotra and Murnighan, 2002; Friedrich and Southwood, 2011). As such, promises are particularly useful in social hierarchies by acting to decrease feelings of distrust between individuals of different social status (Fiske, 2010a; Lount and Pettit, 2012). Promises are ubiquitously used not only to signal/foster trustworthiness to the hierarchy (i.e., pledges or oaths), but are also critical in facilitating trust between individuals of different social ranks, from a high-ranking politician promising voters that she will increase the economy to a low-ranking employee assuring her manager that she will finish her work on time. Despite the importance of promises in facilitating trust between different members of a hierarchy, it is common knowledge that promises are not always kept, and broken promises can have large downstream effects on trust at both personal (Simpson, 2007) and economic levels (Zak and Knack, 2001), making the evaluation of promise outcomes (i.e., promise kept vs. promise broken) of utmost importance to understanding trust in social hierarchies. However, the effects of social status on the evaluation of promise outcomes is far from understood at both behavioral and neural levels.

Previous work on responses to promise outcome evaluation in social hierarchies is almost completely restricted to feedback related to high status promisors (e.g., politicians; Johnson and Ryu, 2010; Corazzini et al., 2014; Born et al., 2017). Here we turn to related work regarding the effects of social status on responses to social norm violations to inform our hypotheses regarding the potential differential effects of lower status and higher status on promise outcome evaluation. In particular, there is an ongoing debate regarding the effects of social status on the evaluation of social norm violations (Wahrman, 2010). One line of research suggests that high status norm violation is judged more harshly than that of their low status counterparts because people tend to have higher expectations of high status than low status others, attributing them with more intentionality and perceiving them as being more responsible for wrongdoing (i.e., “expectation violation” account; Wahrman, 1970; Hamilton and Sanders, 1981). In one study, participants rated the norm violation (i.e., underpayment of personal income taxes) of a wealthy and politically connected New Yorker (i.e., higher status) as being more intentional and recommended increased punishment severity for this norm violation than the same action committed by an immigrant to New York (i.e., low status; Fragale et al., 2009). Moreover, research has found that when a low status (employee) and high status (boss) individual enter into a formal agreement, people are more likely to break off the agreement if the boss breaks the terms of the agreement than if the employee breaks the terms of the agreement (Fiddick and Cummins, 2001).

A second line of research shows that people judge high status norm violation *less* harshly than that of their low status counterparts (Bowles and Gelfand, 2010; von Essen and Ranehill, 2013). In a field study, researchers measured people’s responses to a low or high status individual (based on dress and perceived occupation) who accidentally knocked over a person’s briefcase (Ungar, 1981); they found that while blame assigned to low or high status others was the same, people were more likely to derogate (i.e., judge in a negative way) low status than high status suitcase-kickers. Some researchers speculate that this decreased response to high status norm violations may be because high status individuals are given “idiosyncrasy credits” and “wiggle room” to engage in more creative and more beneficial, but sometimes unethical, behavior because they have more value to add to the group (i.e., “social value” account; Hollander, 1958; Polman et al., 2013). As long as the norm violator has not “used up” her/his credits and retains value to the group, then the norm violation of the high status individual goes unpunished. If, however, high status deviance results in group failure, then high status group members are punished even more severely than low status group members (Wiggins et al., 1965; Alvarez, 1968).

One reason for this divergence in the literature is that the magnitude of the social norm violation is inconsistent across studies. For example, accidentally kicking over one’s briefcase (low magnitude social norm violation) and not paying federal income taxes (high magnitude social norm violation) lead to opposite effects of social status on social norm violation evaluation. Moreover, almost all studies mentioned above are restricted to the evaluation of social norm violations, overlooking the evaluation of social norm *adherence*. Another reason for the divergence in the above-mentioned literature is that the majority of these studies manipulate the socioeconomic status (SES) of the target individual being judged, which has two major disadvantages. The first is that they fail to account for the participant’s own relative social status, which is problematic given that previous research on social status shows that self-status and other-status often interact to affect evaluation of norm violation (Cummins, 1999; Haselhuhn et al., 2015) and social interaction/related processing (Deaner et al., 2005; Ly et al., 2011; Blue et al., 2016). The second disadvantage is that SES is often confounded by feelings of power, making it difficult to distinguish which effects are uniquely driven by social status, and which effects are driven by power. In fact, some researchers speculate that what is driving the diminished punishment of high status norm violators is not “social value” *per se*, but instead a fear of retaliation from the high status norm violators (Homans, 1961; Aquino et al., 2006), which suggests that power may be confounding the effects of social status on norm violation evaluation.

Social status and power are similar but distinct and have been shown to have different effects on behavior and social cognition (Magee and Galinsky, 2008; Blader and Chen, 2012; Dubois et al., 2015; Blader et al., 2016). One common type of social status, socioeconomic status (i.e., SES), is composed of Objective SES and Subjective SES, where Objective SES refers to an individual’s/parents’ salary, vocation, and/or highest achieved level of education (Oakes and Rossi, 2003; Kraus et al., 2009), and Subjective SES refers to an individual’s feelings regarding his/her relative level of salary, vocation, and education in comparison with a relevant population (Adler et al., 2000). In contrast, power (i.e., dominance-based status) refers to an individual’s level of control over another individual’s access to a valued resource or outcomes (Dépret and Fiske, 1993; Galinsky et al., 2003; Keltner et al., 2003; Fiske, 2010b). To disentangle these two types of status constructs, researchers often turn to prestige-based status measures and manipulations (Zink et al., 2008). Prestige-based status refers to the amount of deference, respect, or admiration an individual receives along a relevant domain (Adler et al., 2000; Henrich and Gil-White, 2001; Fiske, 2010b). This type of social status is particularly advantageous because it is distinct from power and wealth and is easily manipulated in a lab setting.

To address the above-mentioned limitations, in the current study we systematically analyze the behavioral and neural effects of both prestige-based status (manipulated at the beginning of the experiment) and SES (measured after the experiment) on promise feedback evaluation. We do so by manipulating participants’ prestige-based status before playing as Investor in a modified version of iterated Trust Game (TG) with promises (Charness and Dufwenberg, 2006, 2010). Participants’ prestige-based status was manipulated via performance ranking on a math quiz in comparison with six confederate players. This is a proven and established inducer of prestige-based status (Hu et al., 2016) with the advantage that it can control for other potential confounds such as power or dominance. In line with previous research (Albrecht et al., 2013), we also control for potential emotional confounds of achieving low-status or high-status ranking (Steckler and Tracy, 2014) by endowing participants with a middle-status ranking in comparison with the six other players and pair them with partners of lower or higher status. After receiving their ranking, participants played several trials of TG as Investor with these players (whose identity was kept anonymous) acting as Trustees. At the beginning of each TG trial, the participant first viewed the social status of the Trustee (lower vs. higher) who had been drawn randomly from the pool of six confederates. To prevent reputation effects and learning, no other personal information was given at this stage. Then, to measure the effects of social status on responses to promise-based feedback, in TG, Trustees either promised (“promise” condition) or did not promise (“no promise” condition, filler) to return half of the multiplied sum (i.e., half of the investment amount after it has been multiplied by 4) to the participant. To create a condition where promise information was not available, in certain trials, Trustees were not given the opportunity to make a promise decision (i.e., “unknown” condition). After viewing the promise information, participants decided whether or not to invest 2 yuan, which was endowed to the investor, in the Trustee. This amount was set at 2 yuan to control for potential magnitude effects of returning or not returning. Finally, the participant was given feedback regarding whether the Trustee had behaved in a trustworthy manner (i.e., return in the “unknown” condition) or in an honest manner (i.e., return in the “promise” condition) before beginning the next trial of TG. In this way, participants experienced both negative *and* positive outcome feedback. Feedback was given regardless of whether or not the participants invested in the Trustee (i.e., forced feedback). This measure was taken to ensure that all participants were made aware that lower and higher status Trustees were trustworthy and honest in half of the trials. We recorded event-related potentials (ERPs) time-locked to the TG feedback. The empirical question was whether and how social status modulates the behavioral and neural responses to honesty and trustworthiness feedback.

At the behavioral level, we focused on the investment rate in TG. Our previous work using a similar prestige-based status manipulation before measuring participants’ behavior as Investor in iterated one-shot TG shows that participants tend to invest more in higher status Trustees than lower status Trustees, and that this effect is most pronounced in the “promise” condition (Blue et al., under review). Given these findings, and considering the two diverging accounts regarding the effects of social status on responses to norm violation, two hypotheses emerge: the “social value” hypothesis would predict that participants would be more likely to invest in higher status promises than in lower status promises, despite feedback showing that lower and higher status partners were equally honest (“social value” hypothesis). The “social value” hypothesis contrasts with an alternative “expectation violation” hypothesis, which would predict that participants would be more surprised by higher status partners’ dishonesty and lack of trustworthiness in 50% of the trials than by that of lower status partners, and would thus invest less in higher status than lower status partners over time.

At the neural level, we focused on two ERP components time-locked to TG feedback which are known for their involvement in outcome evaluation: Medial-frontal negativity (i.e., MFN) and P300 (i.e., P3). The “social value” hypothesis would predict that P300 amplitudes would be more positive-going in response to higher status honesty than lower status honesty, whereas the “expectation violation” hypothesis would predict that MFN responses would be more negative-going to higher status dishonesty than lower status dishonesty. Below, we briefly introduce MFN and P300 and their role in outcome evaluation before we move on to the methodological details of the study.

MFN reflects a family of components related to negative performance feedback (i.e., feedback-related negativity, FRN) and error-related processing (i.e., error-related negativity; ERN). MFN is a negative deflection peaking between 200 and 350 ms post-onset and is found in the frontocentral electrodes. MFN is generated by activity in the anterior cingulate cortex (i.e., ACC). It is often described as reflecting whether the evaluation of events/feedback is good or bad (Gehring and Willoughby, 2002; Yeung and Sanfey, 2004; Sato et al., 2005). In particular, MFN amplitudes are more pronounced for negative feedback and unfavorable outcomes than for positive feedback and favorable outcomes. The reinforcement learning account of MFN states that the mesencephalic dopamine system sends reinforcement learning signals, which are manifest by changes in phasic dopamine (Schultz et al., 1997), via the basal ganglia to ACC, which then learns which decisions are best (Holroyd and Coles, 2002). The MFN thus reflects the reinforcement learning signals to ACC: negative prediction errors (i.e., outcome is worse than expected) elicit greater MFN amplitudes, reflecting decreases of phasic dopamine to ACC, whereas positive prediction errors (i.e., outcome is better than expected) elicit decreased MFN amplitudes, reflecting increases of phasic dopamine to ACC.

Apart from outcome valence, MFN has also been shown to be sensitive to outcome expectancy (Jia et al., 2007; Wu and Zhou, 2009), such that outcomes that are less expected elicit a more pronounced MFN response. Expectancy violation effects on MFN are also found in social contexts (Wu et al., 2011b), such as social norm violations. MFN is sensitive to social norm violations, such as those related to fairness and generosity (Boksem and De Cremer, 2010; Wu et al., 2011a). Violation of trust, another form of social norm violation, also elicits enhanced MFN responses (Long et al., 2012). Moreover, relevant to the current study, previous research also shows that MFN is sensitive to Trustee honesty in TG (Ma et al., 2015). A few studies have found that social factors, such as social distance (Ma et al., 2011; Wu et al., 2011a), can modulate MFN responses to norm violations, suggesting that factors such as social status may also be capable of modulating the effect of MFN on TG feedback. If higher status dishonesty elicits greater expectation violation (i.e., is perceived as a greater social norm violation) than lower status dishonesty, then the MFN effect should be more pronounced for higher status than lower status dishonesty.

P300 (also referred to as P3; Sutton et al., 1965) represents later top-down attentional resources devoted to outcome evaluation and reward. P300 is the most positive peak 200–600 ms post-onset of feedback and is found in the medial parietal electrodes. The P300 was originally recognized as encoding the motivational significance of a stimulus (Duncan-Johnson and Donchin, 1977). Motivationally significant stimuli are those that “are either relevant to the current task or that have the potential to be associated with some form of utility (positive or negative)” (Nieuwenhuis et al., 2005, p. 511). In addition to the motivational significance of the stimulus, P300 is modulated by the probability of a stimulus occurrence (Donchin and Coles, 1988) and the amount of attention paid to the stimulus (Yeung et al., 2005; Wu and Zhou, 2009).

P300 is also involved in outcome evaluation. Certain studies measuring P300 responses to gambling outcomes have shown that P300 is sensitive to the magnitude of the outcome, but not its valence (Yeung and Sanfey, 2004; Sato et al., 2005; Yeung et al., 2005), causing these researchers to attribute P300 as being sensitive only to the motivational significance of an outcome. However, other research on P300 responses to gambling outcomes shows that P300 amplitude is sensitive to both the magnitude and the valence of the outcome (Hajcak et al., 2005, 2007; Wu and Zhou, 2009), suggesting that P300 reflects broad cognitive appraisal processing related to attention and reward (Gray et al., 2004; Linden, 2005). P300 amplitudes are also sensitive to social factors in outcome-processing (Li et al., 2010; Zhou et al., 2010; Ma et al., 2011). One study showed that when gambling outcomes were compared with those of friends and strangers, P300 was sensitive not only to the reward valence, but also to the social distance of the person with whom they were comparing gambling outcomes (Leng and Zhou, 2010), which suggests that social factors can modulate the effect of attention or motivational significance on P300.

In trust-related situations, P300 may also reflect social value above and beyond simple trustworthiness feedback. For example, Boudreau et al. (2009) measured participants’ trust behavior in a coin-toss game, in which participants guessed whether a coin tossed by a partner was heads or tails based on the partner’s indication. In one condition, participants had common interests with the partners (i.e., if the participant guessed correctly, they both received a monetary reward); in another condition, participants were told that partners would receive a penalty for misleading the participant. Findings showed that, despite the equal probability of participants trusting partners in the “common interests” and “penalty for lying” conditions (96 and 97%, respectively), participants’ P300 responses were more pronounced for recommendations given by partners with common interests than by partners who would receive a penalty for lying, which suggests that other factors, such as social value, may modulate P300 above and beyond simple perceived trustworthiness levels.

Similar work using TG shows that factors related to the Trustee can influence investment behavior in the Trustee and neural responses to Trustee TG outcome feedback (i.e., “return” vs. “no return”; Delgado et al., 2005). In particular, Fareri et al. (2015) found that participants acting as Investors were more likely to invest in Trustees who were their friends than Trustees who were strangers, and that activity in ventral striatum, a brain region known for reward processing, was greater when friends returned than when strangers returned, despite the fact that the reinforcement rate (i.e., return percentage) was equal for friends and for strangers. The authors found support showing that the neural and behavioral responses were influenced by the increased “social value” of the Trustee when they were friends, compared with when they were strangers (Fareri et al., 2015). Similarly, when viewing TG outcomes, return decisions by Trustees with good reputations elicit greater activity in ventral striatum than the same return responses from partners with bad reputations (Phan et al., 2010), which further demonstrates that certain Trustee characteristics may increase the perceived reward of return decisions in TG. Moreover, work simultaneously measuring fMRI and EEG during gain and loss anticipation shows that increased P300 amplitudes for gains over losses is positively correlated with ventral striatum activity during the anticipation of gains (Pfabigan et al., 2014), which suggests that P300 reward processing may be associated with reward processing in ventral striatum. Taken together, we suspect that P300 will not only detect the valence of TG feedback (i.e., “return” vs. “no return”), but that factors potentially related to perceived “social value,” such as social status and honesty, may also influence attentional resources devoted to TG feedback by interacting with P300 during TG outcome processing.

Finally, we test for the potential effects of SES on the evaluation of TG outcomes. SES has been shown to modulate attention allocation in social settings (Dietze and Knowles, 2016). Moreover, past research shows that, in comparison with lower status, interaction with higher status others elicits greater activity in the ventral striatum (Zink et al., 2008), and that activity in the ventral striatum can be modulated by participants’ SES when viewing low and high status others (Ly et al., 2011). Research on monkeys also shows that rhesus macaques will give up highly valued rewards (i.e., sugary liquid) in order to view high status others, and that this effect is modulated by rhesus macaques’ own social status (Deaner et al., 2005). Taken together, we suspect that participants’ SES may modulate the valuation of lower and higher status honesty.

## Materials and Methods

### Participants

To determine the sample size, we used G^∗^Power 3 software (Faul et al., 2007), which showed that we needed a sample size of at least 32 for this study to have adequate power (1 – β > 0.95) to detect a medium-size effect (*f* = 0.30). The power analysis (repeated-measures, within participants effect) was performed for the interaction between partner social status (lower vs. higher) and promise (promise vs. unknown). The correlation among repeated measures was set at 0.6, which was based off of the correlation among repeated measures in a previous behavioral pilot study [*r*(28) = 0.594; Blue et al., under review]. Among the 42 participants we tested, two were removed because alpha-wave artifacts, two failed the post-experiment questionnaire for understanding the experimental setup and task requirements, four were suspicious of the experimental setup, and one was removed due to a technical malfunction. These nine participants were removed from data analysis, leaving 33 participants (20 females) in the following analysis whose age was between 18 and 23 years (mean: 19.70 years, *SD* = 1.40). All participants were healthy, right-handed, had normal or corrected-to-normal vision, and no participants had a history of neurological or psychiatric disorders. Before the experiment, all participants gave their informed consent and were informed that the basic payment for participation was 80 Chinese yuan (about 12.5 USD) with a bonus of 0–15 yuan, which was based on performance in TG. The experiment was in accordance with the Declaration of Helsinki and was approved by the Ethics Committee of the School of Psychological and Cognitive Sciences, Peking University.

### Design and Procedure

The experiment had a 2 (partner social status: lower vs. higher) × 2 (promise: promise vs. unknown) × 2 (return: return vs. no return) within-participants factorial design. An additional filler condition, in which the partner had an opportunity to promise but did not choose to promise (“no promise” condition) was included to increase the perceived agency in the “promise” condition. As in past experiments (Hu et al., 2014, 2016), we used a star system (Zink et al., 2008) to assign social status, with one star indicating low status, two stars indicating middle status, and three stars indicating high status. The investment decision was binary (i.e., invest vs. no invest). The investment amount was set at 2 yuan, making the multiplied sum 8 yuan.

**FIGURE 1:**
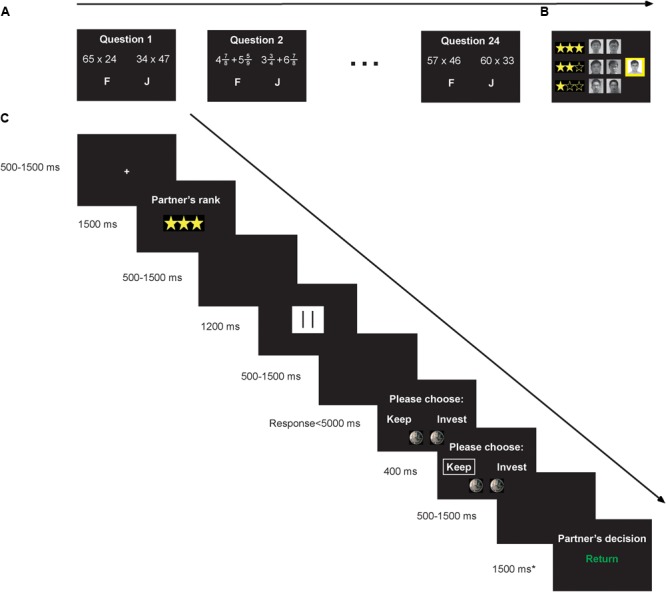
Schematic diagram of the experiment. **(A)** In the rank-inducing task (i.e., math quiz), participants were given 10 s to select which of two arithmetic expressions had a greater value by pressing the “F” or “J” key with the left or right index finger. **(B)** Upon completion of the quiz (24 problems in total), participants were shown their performance ranking in comparison with six same-sex confederate players. All participants were assigned a middle (2-star) ranking, highlighted in yellow. **(C)** In TG, participants acted as Investor with Trustees of lower and higher status. On each trial, participants viewed the Trustee’s ranking from the math quiz along with his/her promise information before deciding whether or not to invest in the Trustee. Regardless of whether the participant invested in the Trustee, participants were shown the return feedback. Return feedback was set at 50% regardless of the condition. We focused on the ERP responses time-locked to the feedback screen (noted with the asterisk).

Participants arrived alone to the laboratory for each experimental session, where they were told that six same-sex participants (confederates) were ostensibly waiting in another room. Participants then gave permission to have their photo taken, which would later be used in the math quiz ranking screen, along with the photos of the six confederates. The participants were told that the six confederates would also complete the math quiz and would later act as their partner in TG (**Figure 1**).

The math quiz task is an established inducer of social status (Hu et al., 2016). Participants were given 10 s to select which of two arithmetic expressions had a greater value by pressing the “F” or “J” key with the left or right index finger. If the participant had not selected a response after 7 s, he/she was given a reminder that time was running out on that particular question. Each problem was composed of either two-digit multiplication (e.g., 45^∗^72) or complex fraction addition (e.g., 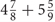). In total, there were 24 arithmetic problems (12 easy, 12 difficult). Half of the problems were solvable in the time allotted while the other half were extremely difficult to solve in the time allotted, which facilitated the participant’s belief that they had achieved a two-star (middle status) ranking. Upon completion of the quiz, participants viewed their rank in comparison with the ranks of the six other confederates. All participants were assigned a middle (2-star) ranking in order to avoid the potential influences of emotion after gaining high or low status (Steckler and Tracy, 2014) and to test the effects of others’ social status on participants’ responses to promise outcome feedback (Albrecht et al., 2013).

In TG, participants acted as Investor, and the six confederates from the math quiz acted as Trustees. Participants were informed that they would only be paired with Trustees who had achieved rankings that were different from their own, so they only faced low (one-star) and high (three-star) status Trustees. This was meant to increase the number of trials in the critical conditions. At the start of each trial (264 trials in total), participants were given 2 yuan. Then, participants viewed the ranking of their anonymous partner for that particular trial. Next, participants viewed the partner’s promise decision, with “ ! ” indicating that the partner promised to return 4 yuan (50% of the multiplied sum; “promise” condition), “- -” indicating that the partner did not promise to return 4 yuan (“no promise” condition; filler), and “ || ” indicating that the partner was not given the opportunity to make a promise decision on that trial (“unknown” condition). Then the participant chose whether or not to invest the 2 yuan in the partner. The participant was given a maximum of 5 s and used the “F” and “J” keys on the keyboard to make this decision (“invest” and “keep” decision locations were counterbalanced over trials). If the participant did not make an investment decision within 5 s, the trial started again from the beginning. If the participant chose to invest the 2 yuan, then the partner received 8 yuan; if the participant chose to keep the 2 yuan, then the partner came away from that trial with nothing. Finally, the participant viewed the Trustee’s feedback (i.e., decision to return or not to return) on that trial. Importantly, participants were told that Trustees made their return decisions at the same time as participants were making their decision to invest or not (i.e., before viewing the participant’s investment decision). This point was emphasized to the participants because it was necessary to give feedback to the participants on each TG trial regardless of whether they invested or not. We used forced feedback for two reasons: (1) to ensure that lower and higher status partner trustworthiness and honesty levels were identical (i.e., lower status Trustees and higher status Trustees both returned on 50% of the “promise” trials and 50% of the “unknown” trials), and (2) to ensure that there were enough trials in the critical conditions for ERP data analysis. As filler trials, we also included certain trials where Trustees did not promise to return in 50% of the multiplied sum (i.e., “no promise” condition; 12 trials in total); in these trials, TG partners did not return.

Each trial of TG began with a fixation sign (white cross subtended 0.3° of visual angle) for either 500, 700, 900, 1100, 1300, or 1500 ms against a black background (**Figure 1C**). On the next screen, participants viewed the words “Your partner’s rank:” in Chinese (white and Song font, size 32) above the star ranking (subtended 2° × 0.8°) for 1500 ms; the star ranking was composed of either a yellow filled star with two empty yellow stars (one-star, lower status rank) or three yellow filled stars (three-star, higher status rank). After the presentation of a blank screen for a jittered time between 500 and 1500 ms, participants then viewed the partner’s promise information for 1200 ms. After the presentation of a blank screen for a jittered time between 500 and 1500 ms, the participants then viewed the words “Please choose” above the choices “Invest” and “Keep” (the locations of which were counterbalanced across trials) for a maximum of 5000 ms. After making their selection, a white box immediately highlighted the answer response for 400 ms. After the presentation of a blank screen for a jittered time between 500 and 1500 ms, the participants finally viewed the words “Partner’s decision:” above the words “Return” or “No return” in Chinese in green and red, respectively, with colors counterbalanced across participants. The final screen appeared for 1500 ms.

EEG data were recorded throughout the experiment. We focused our analysis on the TG feedback screen. The participants were comfortably seated in a dimly lit and electromagnetically shielded room about 1.5 m in front of a computer screen. The experiment used Presentation software (Neurobehavioral System Inc.) to control the timing and presentation of stimuli and was displayed on a Visuosonic 22-in. CRT display. The experiment consisted of the status-inducing task (i.e., math quiz, 24 problems in total) followed by six blocks of TG (44 TG trials per block). There were 30 trials per condition (lower status “unknown” return; lower status “unknown” no return; lower status “promise” return; lower status “promise” no return; higher status “unknown” return; higher status “unknown” no return; higher status “promise” return; higher status “promise” no return) and 24 “no promise” filler trials (12 lower status; 12 higher status) in which Trustees did not return on any trial.

Before beginning the experiment, participants were told that bonus payments (0–15 yuan) were based on TG behavior from 10 randomly selected trials. In addition, all participant completed practice math quiz problems and TG trials until they were comfortable with the setup, with a minimum of 6 math problems and 10 TG trials in the practice session. Participants were also tested on their recognition of the promise symbols. No participants were allowed to begin the experiment without being able to consistently and accurately identify each symbol. No participants reported difficult with remembering the promise symbols.

After the experiment, participants reported on a 7-point Likert scale to what extent they felt superior or inferior (1 = very inferior; 7 = very superior) when facing partners of the two ranks. This measure served as a manipulation check of social status; each participant indicated this rating once for each social status. As an additional manipulation check, participants indicated their own Subjective SES and the Subjective SES of both lower and higher status partners. Subjective SES was measured using the MacArthur Subjective Social Status Scale (Adler et al., 2000), which asks participants to indicate the target’s subjective status in Chinese society on a ladder, with the lowest rungs indicating individuals with the lowest level of income, occupation, and education, and the highest rungs indicating individuals with the highest level of income, occupation, and education. To test for potential effects of Objective SES, participants also indicated their parents’ highest level of education (1 = middle school diploma; 2 = high school diploma/middle trade school certificate; 3 = trade school certificate; 4 = bachelor’s degree; 5 = graduate degree) and their parents’ annual income (1 = 0 – 10,000 yuan; 2 = 10,000 – 100,000 yuan; 3 = 100,000 – 300,000 yuan; 4 = 300,000 – 500,000 yuan; 5 = 500,000 – 1,000,000 yuan; 6 = 1,000,000 – 5,000,000 yuan; 7 ≥ 5,000,000 yuan. Note, for the sake of privacy, participants were allowed to select “8” which indicated that they did not want to respond to this question).

To test for potential differences in learning of lower and higher status trustworthiness and honesty, immediately after the experiment, participants were asked to recall lower and higher status behavior during TG and indicate the ratio of lower status and higher status trustworthiness (percentage of “return” decisions in the “unknown” condition) and honesty (percentage of “return” decisions in the “promise” condition). For each item, participants were asked to indicate any number from 0 to 100%. Finally, to more explicitly measure perceived trustworthiness, participants were asked to indicate perceived trustworthiness of lower/higher status partners based on their behavior in TG. To measure perceived trustworthiness of lower/higher status partners, after the experiment, we recorded participants’ feelings of perceived ability, benevolence, and integrity of lower and higher status TG partners, which are three fundamental components of trustworthiness (Mayer et al., 1995). The perceived trustworthiness measures were the same measures as those used in similar research (Lount and Pettit, 2012), which were drawn from previous work in organizational psychology on trustworthiness perception (Mayer and Davis, 1999). The questions are aimed at addressing employees’ feelings toward employers (“top management”); we adjusted the questions to be less work-oriented and more suitable for students. Participants rated each status of partners on each of the three dimensions. Ability was composed of 6 items (e.g., This individual is very efficient) (α = 0.822); Benevolence was composed of five items (e.g., “This individual is concerned about my welfare.”) (α = 0.805); Integrity was composed of six items (e.g., This individual has a strong sense of justice) (α = 0.798). Participants recorded their responses using a 7-point Likert scale (1 = completely disagree, 7 = completely agree).

### EEG Recording and Analysis

EEGs were recorded from 64 scalp sites using tin electrodes mounted in an elastic cap (Brain Products, Munich, Germany) according to the international 10–20 system. The horizontal electrooculogram (HEOG) was recorded from electrodes placed at the outer cantus of the left eye, and the vertical EOG (VEOG) was recorded supra-orbitally from the right eye. All EEGs and EOGs were referenced online to an external electrode on the tip of the nose; they were re-referenced off-line to the mean of the left and right mastoids. For all electrodes, electrode impedance was kept below 5 kΩ. Bio-signals were amplified with a band-pass from 0.016 to 100 Hz and digitized online with a sampling frequency of 500 Hz.

Offline, we extracted separate EEG epochs (200 ms pre-stimulus to 800 ms post-stimulus), which were time-locked to the onset of the TG feedback screen. The EEG data were high-pass filtered at 0.1 Hz and low-pass filtered at 30 Hz. Baseline correction for each epoch was done by subtracting the average activity of the channel during the baseline period from each sample. Trials in which EEG voltages exceeded ± 70 μV were excluded from further analysis. After artifact rejection, an average of 86% of trials (*SD* = 10%) of the epochs on the TG feedback screen were entered into statistical analysis.

For statistical analysis, electrodes were divided based on two three-level factors: Region (anterior vs. central vs. posterior) and Hemisphere (left vs. medial. vs. right), which resulted in 9 regional clusters: the left anterior cluster was composed of F3, F5, FC3, and FC5; the medial anterior cluster was composed of F1, Fz, F2, FC1, FCz, and FC2; the right anterior cluster was composed of F4, F6, FC4, and FC6; the left central cluster which was composed of C3, C5, CP3, and CP5; the medial central cluster was composed of C1, Cz, C2, CP1, CPz, and CP2; the right central cluster was composed of C4, C6, CP4, and CP6; the left posterior cluster was composed of P3, P5, and PO7; the medial poster cluster was composed of P1, Pz, P2, PO3, POz, and PO4; the right posterior cluster was composed of P4, P6, and PO8. This clustering method for analyzing the EEG data is similar to the method used in a related study analyzing P300 in response to feedback in a social dilemma game (Bell et al., 2016). For statistical purposes, we averaged the amplitude and/or peaks over electrodes in each regional cluster. Time windows were determined by visual inspection of the waveforms and preliminary analyses.

For ERP responses to the TG feedback, we focused our analysis on MFN (the mean amplitudes in the time window of 250–310 ms) and P300 (the peak values in the time window of 250–600 ms). For MFN, we focused our analysis on the medial anterior cluster. We selected these electrodes because the MFN effect was largest on these electrodes. We conducted ANOVA with three within-subjects factors: partner social status (lower vs. higher), promise condition (promise vs. unknown), and return (return vs. no return). For P300, we conducted ANOVAs with five within-subjects factors: partner social status (lower vs. higher), promise condition (promise vs. unknown), return (return vs. no return), region (anterior vs. central vs. posterior), and hemisphere (left vs. medial vs. right). In order to account for multiple comparisons, Bonferroni correction was used when appropriate. In cases of non-sphericity, we applied the Greenhouse–Geisser correction.

## Results

### Manipulation Check of Social Status

In order to ensure that the social status manipulation elicited feelings of inferiority and superiority, we conducted a one-factor (star-ranking: one vs. three) repeated-measures ANOVA, which confirmed the social status manipulation, *F*(1,31) = 57.923, *p* < 0.001, $\eta_{p}^{2}$ = 0.651. Participants reported higher feelings of superiority when facing a lower status partner (5.313 ± 0.171) than when facing a higher status partner (3.469 ± 0.149). The status manipulation also affected feelings of Subjective SES, *F*(2,64) = 39.123, *p* < 0.001, $\eta_{p}^{2}$ = 0.550, as participants rated three-star partners as having a higher Subjective SES (6.955 ± 0.224) than their own (6.015 ± 0.250), *p* < 0.001, and one-star partners as having lower Subjective SES (5.045 ± 0.231) than their own (6.015 ± 0.250), *p* < 0.001. Objective SES results are reported below (see *Objective SES*).

### Behavioral Results

**FIGURE 2:**
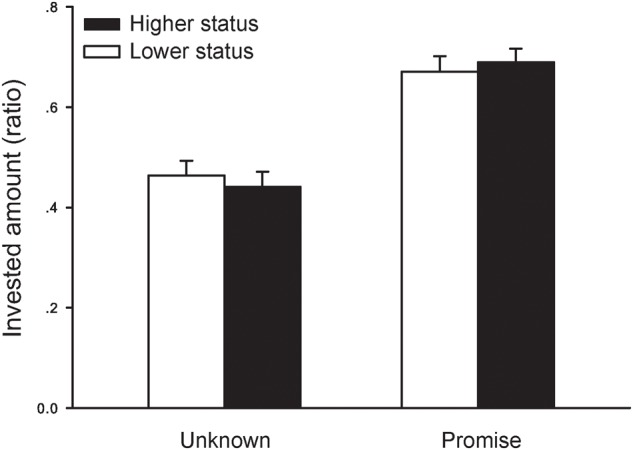
Mean and standard error of the means for ratio of investment in partners of different social status across the two promise conditions (“unknown” vs. “promise”).

A repeated-measures analysis of variance (ANOVA) showed that the investment ratio varied as a function of promise, *F*(1,32) = 52.019, *p* < 0.001, $\eta_{p}^{2}$ = 0.619 (**Figure 2**). Participants were more likely to trust (i.e., invest) in “promise” trials (mean ± SE, 0.681 ± 0.027) than in “unknown” trials (0.453 ± 0.027). There was no main effect of partner social status, *p* = 0.915. Importantly, consistent with our previous studies (Blue et al., under review), there was a non-significant trend or tendency of an interaction between partner social status and promise conditions, *F*(1,32) = 3.783, *p* = 0.061, $\eta_{p}^{2}$ = 0.106. Further tests revealed that when interacting with higher status partners, participants tended to be more likely to invest in “promise” trials (0.69 ± 0.03) than in “unknown” trials (0.44 ± 0.03, *p* < 0.001, $\eta_{p}^{2}$ = 0.64), and this effect was smaller for participants when playing with lower status partners (“promise” condition: 0.67 ± 0.03, “unknown” condition: 0.46 ± 0.03, *p* < 0.001, $\eta_{p}^{2}$ = 0.54).

To evaluate the strength of the empirical evidence in favor of (or against) the interaction between partner social status and promise conditions, we also conducted a Bayes factor analysis (Dienes, 2014). Bayes factor analysis tests the strength of evidence between two theories (a null hypothesis theory and the proposed effect in the data), and its value ranges from 0 to infinity, with an increase in value indicating stronger support to reject the null hypothesis. The conventional cut-offs for Bayes factor sensitivity are 1/3 and 3, which means that any value outside of this range (less than 1/3 or greater than 3) provides strong evidence in support of the null hypothesis or the proposed effect in the data, respectively. Values between 1/3 and 3 are considered weak or “anecdotal” evidence (Jeffreys, 1939/1961). Our analysis was conducted using the BayesFactor (Morey et al., 2015) package in the R statistical language. We found a Bayes factor of 2.508 ± 7.65% which suggests that there is an interaction between partner social status and promise condition, but that it is a weak effect. This result indicates that independent confirmation is needed to confirm the interaction between partner social status and promise conditions.

To examine potential differences in learning of lower and higher status trustworthiness and honesty, after the experiment we tested participants’ recall of lower and higher status trustworthiness (i.e., ratio of return in the “unknown” condition) and honesty (i.e., ratio of return in the “promise” condition). In particular, to measure recall of trustworthiness, we analyzed participants’ responses to the prompts: “Please indicate what percentage of the time (lower) higher status partners returned half of the multiplied sum when they did not have an opportunity to promise to do so?”; to measure recall of honesty, we analyzed participants’ responses to the prompts: “Please indicate what percentage of the time (lower) higher status partners returned half of the multiplied sum when they promised to do so?” There was no difference in recall of lower status and higher status trustworthiness, *t*(32) = 0.376, *p* = 0.709, or honesty, *t*(32) = 1.491, *p* = 0.146. As an additional check of learning, we entered the difference between higher and lower status honesty investment for each block (i.e., [(higher status “promise” – higher status “unknown”) – (lower status “promise” – lower status “unknown”)]) into a one-factor (block: 1 vs. 2 vs. 3 vs. 4 vs. 5 vs. 6) repeated-measures ANOVA, which was not significant, *F* < 1; *p* = 0.997. We also conducted this one-factor repeated-measures ANOVA separately for status differences in each block of the “unknown” condition, *F* = 1.571, *p* = 0.171, and each block of the “promise” condition, *F* = 1.491, *p* = 0.195. Regardless of the condition, our data show that participants’ investment behavior showed no evidence of changing over time. Taken together, these results indicate that there is no evidence that participants learned or adjusted their behavior across the experiment.

Results regarding the post-experiment perceived trustworthiness measurements (i.e., ability, benevolence, and integrity) were as follows. Participants rated higher status partners (4.697 ± 0.117) as having greater ability than lower status partners (4.066 ± 0.110), *t*(32) = -4.937, *p* < 0.001. There was a non-significant trend or tendency for participants rating higher status partners (3.042 ± 0.149) as being more benevolent than lower status partners (3.430 ± 0.187), *t*(32) = 1.954, *p* = 0.059. There was no difference in participants’ ratings of higher status (3.859 ± 0.147) and lower status (4.015 ± 0.164) partner integrity, *p* = 0.473. We also tested for the possibility that differences in these factors between lower and higher status may have correlated with the TG behavior interaction between partner social status and promise conditions [i.e., (Higher status “promise” – Higher status “unknown”) – (Lower status “promise” – Lower status “unknown”)]. No evidence was found for the role of perceived ability, benevolence, or integrity to predict the behavioral interaction between partner social status and promise conditions, perceived ability, *p* = 0.699; perceived benevolence, *p* = 0.276; perceived integrity, *p* = 0.569.

### MFN in the 250–310 ms Time Window Following TG Feedback

**FIGURE 3:**
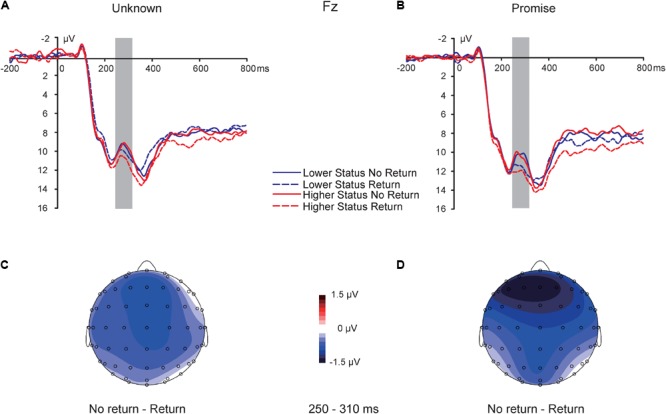
Medial frontal negativity (MFN). ERP response time-locked to the onset of the TG return feedback at the Fz electrode in the “unknown” **(A)** and “promise” **(B)** conditions. Topographic maps for the MFN effect in the 250–310 ms time window in the “unknown” **(C)** and “promise” **(D)** conditions.

For ERPs time-locked to the TG feedback (**Figure 3**), in the time window of 250–310 ms in the medial anterior cluster of electrodes, a 2 (partner social status: lower vs. higher) × 2 (promise: promise vs. unknown) × 2 (return: return vs. no return) repeated-measures ANOVA showed a significant main effect of return *F*(1,32) = 6.147, *p* = 0.019, $\eta_{p}^{2}$ = 0.161, indicating that participants evidenced more negative-going MFN in response to “no return” feedback (10.738 ± 0.982 μV) than to “return” feedback (11.803 ± 0.931 μV). There was also a significant main effect of promise *F*(1,32) = 12.747, *p* = 0.001, $\eta_{p}^{2}$ = 0.285, indicating that feedback in the “unknown” condition elicited more negative-going MFN (10.755 ± 0.912 μV) than feedback in the “promise” condition (11.787 ± 0.974 μV). There was no main effect of partner social status, *p* = 0.289. Moreover, there was no interaction between the three conditions (interaction between promise and partner social status, *p* = 0.982; interaction between promise and return, *p* = 0.346; interaction between partner social status and return, *p* = 0.308; interaction between partner social status, promise, and return, *p* = 0.741).

Given that forced feedback was given to the participant regardless of whether or not an investment decision was made, we tested for the potential effect of investment on MFN response. To test whether MFN responses were modulated by investment behavior, we compared average MFN responses from the same medial anterior cluster electrodes in the 250–310 ms time window time-locked to the TG feedback. In particular, we compared MFN amplitudes from only those trials in which participants invested (i.e., invest-only trials) with MFN amplitudes on all trials regardless of investment (i.e., all trials). Given the limited number of trials (30 trials/condition) and given that participants only invested in 45% of the trials in the “unknown” condition, we only analyzed trials from the “promise” condition (participants invested on 68% of “promise” condition trials). There were too few no-invest trials in both the “promise” and the “unknown” conditions to conduct a meaningful comparison between invest-only trials and no-invest trials, thus we compared invest-only trials with all trials. After removing “promise” condition trials in which the participant did not invest, 7 participants had less than 15 trials per condition. These 7 participants were removed from this supplementary analysis, leaving 26 participants in the analysis. A 2 (invest: yes vs. all trials) × 2 (partner social status: lower vs. higher) × 2 (return: return vs. no return) repeated-measures ANOVA revealed a main effect of return, *F*(1,25) = 5.053, *p* = 0.034, $\eta_{p}^{2}$ = 0.168, indicating that participants evidenced more negative-going MFN in response to “no return” feedback (10.133 ± 0.997 μV) than to “return” feedback (11.539 ± 0.972 μV). There was a significant interaction between invest and return, *F*(1,25) = 6.891, *p* = 0.015, $\eta_{p}^{2}$ = 0.216. Further tests showed that the main effect of return was stronger for invest-only trials, *F*(1,25) = 6.692, *p* = 0.016, $\eta_{p}^{2}$ = 0.211, than in trials that included both invest and no invest trials, *F*(1,25) = 2.973, *p* = 0.097, $\eta_{p}^{2}$ = 0.106. No other effects reach significance.

### P300 Following TG Feedback

**FIGURE 4:**
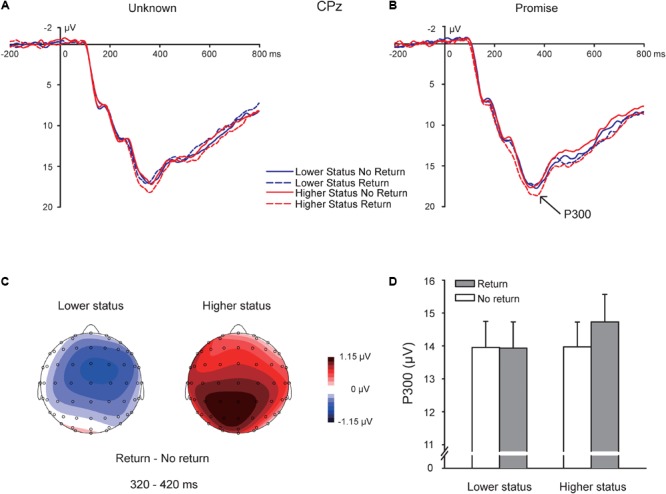
P300. ERP response time-locked to the onset of the TG return feedback at the CPz electrode in the “unknown” **(A)** and “promise” **(B)** conditions. Topographic maps for the P300 effect in the 320–420 ms time window **(C)**. Bar plot depicting the whole-brain average peak values for P300 responses to “return” and “no return” feedback for lower status and higher status partners **(D)**.

The peak amplitudes of the P300 time-locked to the TG feedback (**Figure 4**) were entered into a 2 (partner social status: lower vs. higher) × 2 (promise: promise vs. unknown) × 2 (return: return vs. no return) × 3 (region: anterior vs. central vs. posterior) × 3 (hemisphere: left vs. medial vs. right) repeated-measures ANOVA. There was a significant main effect of partner social status, *F*(1,32) = 6.345, *p* = 0.017, $\eta_{p}^{2}$ = 0.165, indicating that feedback related to higher status partners evoked a more positive-going P300 amplitude (14.351 ± 0.779 μV) than feedback related to lower status partners (13.945 ± 0.780 μV). There were no significant main effects of promise, *p* = 0.142, or return, *p* = 0.172. The interaction between partner social status, promise, and return was not significant, *p* = 0.632. Interestingly, the interaction between partner social status and return was significant, *F*(1,32) = 4.819, *p* = 0.036, $\eta_{p}^{2}$ = 0.131, such that P300 amplitudes were more positive-going for higher status “return” feedback (14.731 ± 0.833 μV) than higher status “no return” feedback (13.972 ± 0.752 μV), *p* = 0.019, while there was no difference in P300 amplitude for lower status return feedback (lower status “return:” 13.934 ± 0.798 μV; lower status “no return:” 13.955 ± 0.796 μV, *p* = 0.949) (**Figure 4D**). Moreover, P300 amplitudes were more positive-going in response to higher status “return” feedback (14.731 ± 0.833 μV) than to lower status “return” feedback (13.934 ± 0.798 μV), *p* = 0.001, while there was no difference in P300 amplitudes in response to “no return” feedback between higher status partners (13.972 ± 0.752 μV) and lower status partners (13.955 ± 0.796 μV), *p* = 0.949. If we restrict our analysis to the peak values on the electrode CPz, and enter these peak values into a 2 (partner social status: lower vs. higher) × 2 (promise condition: promise vs. unknown) × 2 (return: yes vs. no) repeated-measures ANOVA, the same pattern of effects was obtained, with the exception that there was a non-significant trend or tendency for a main effect of status, *F*(1,32) = 3.763, *p* = 0.061, $\eta_{p}^{2}$ = 0.105.

Regarding the influence of the electrode location, there was a main effect of hemisphere, *F*(2,64) = 115.938, *p* < 0.001, $\eta_{p}^{2}$ = 0.784, with P300 amplitudes being most positive-going in the medial hemisphere (16.866 ± 0.927 μV) than in the left (12.409 ± 0.721 μV) and right (13.170 ± 0.721 μV) hemispheres, *p*s < 0.001. P300 amplitudes were also more positive-going in the right hemisphere than in the left hemisphere, *p* = 0.017. There was a main effect of region, *F*(2,64) = 15.891, *p* < 0.001, $\eta_{p}^{2}$ = 0.332, with P300 amplitudes being more positive-going in the central region (16.006 ± 0.896 μV) than in the anterior (13.600 ± 0.907 μV) and posterior (12.839 ± 0.722 μV) regions, *p*s < 0.001. Importantly, there was a significant interaction between partner social status and region, *F*(2,64) = 3.764, *p* = 0.048, $\eta_{p}^{2}$ = 0.105, such that higher status feedback elicited a more positive-going P300 than lower status feedback in the anterior region (lower status: 13.322 ± 0.907 μV; higher status: 13.878 ± 0.919 μV, *p* = 0.015) and the central region (lower status: 15.754 ± 0.897 μV; higher status: 16.257 ± 0.906 μV, *p* = 0.011), whereas in the posterior region, there was no difference in P300 amplitude for lower and higher status feedback (lower status: 12.758 ± 0.739 μV; higher status: 12.920 ± 0.711 μV, *p* = 0.273). There was also an interaction between promise and region, *F*(2,64) = 7.814, *p* = 0.004, $\eta_{p}^{2}$ = 0.196, such that, in the anterior region, feedback in the “promise” condition elicited a more positive-going P300 amplitude (13.986 ± 0.942 μV) than feedback in the “unknown” condition (13.214 ± 0.899 μV), *p* = 0.022, whereas there was no difference in P300 amplitudes for “promise” and “unknown” conditions in the central region (“unknown”: 15.815 ± 0.899 μV; “promise”: 16.196 ± 0.922 μV, *p* = 0.239) or the posterior region (“unknown”: 12.787 ± 0.732 μV; “promise”: 12.891 ± 0.731 μV, *p* = 0.665).

There was a significant interaction between promise, return, and hemisphere, *F*(2,64) = 4.140, *p* = 0.033, $\eta_{p}^{2}$ = 0.115. In the “unknown” condition, there was a significant interaction between return and hemisphere, *F*(2,64) = 5.314, *p* = 0.009, $\eta_{p}^{2}$ = 0.142, whereas in the “promise” condition this interaction was not significant, *p* = 0.974. Tests for simple effects showed that, in the “unknown” condition, feedback indicating “no return” elicited more positive-going P300 amplitudes in the medial hemisphere (16.399 ± 0.902 μV) than in the left hemisphere (11.988 ± 0.699 μV) and right hemisphere (12.956 ± 0.699 μV), *p*s < 0.001; moreover, feedback indicating “no return” also elicited more positive-going P300 amplitudes in the right hemisphere (12.956 ± 0.699 μV) than in the left hemisphere (11.988 ± 0.699 μV), *p* = 0.012. Similarly, feedback indicating “return” elicited more positive-going P300 amplitudes in the medial hemisphere (16.806 ± 0.988 μV) than in the left hemisphere (12.508 ± 0.779 μV) and right hemisphere (12.974 ± 0.787 μV), *p*s < 0.001. There was no difference in P300 amplitudes in the left hemisphere and right hemisphere, *p* = 0.583.

To test whether P300 responses were modulated by investment behavior, we compared peak P300 responses from the medial posterior cluster electrodes time-locked to the TG feedback. In particular, we compared P300 peak amplitudes on only those trials in which participants invested (i.e., invest-only trials) with P300 peak amplitudes on all trials regardless of investment (i.e., all trials). Similar to the MFN analysis, we only analyzed trials from the “promise” condition. There were too few no-invest trials in both the “promise” and the “unknown” conditions to conduct a meaningful comparison between invest-only trials and no-invest trials, thus we compared invest-only trials with all trials. After removing “promise” condition trials in which the participant did not invest, 7 participants had less than 15 trials per condition. These 7 participants were removed from this supplementary analysis, leaving 26 participants in the analysis. A 2 (invest: yes vs. all trials) × 2 (partner social status: lower vs. higher) × 2 (return: return vs. no return) repeated-measures ANOVA revealed a main effect of invest, *F*(1,25) = 10.847, *p* = 0.003, $\eta_{p}^{2}$ = 0.303, indicating that participants evidenced more positive-going P300 when receiving feedback on invest-only trials (14.856 ± 0.949 μV) than on invest and no invest trials combined (14.436 ± 0.916 μV). No other effects reach significance.

### Objective SES

Objective SES was measured using parents’ highest attained level of education and parents’ combined annual salary. Parents’ highest level of education, (*M* = 2.758, *SE* = 0.200) on average ranged from high school diploma/middle trade school certificate to trade school certificate (a level slightly lower than a bachelor’s degree); parents’ average annual salary (*M* = 2.533, *SE* = 0.115) on average ranged from 10,000 yuan to a little over 100,000 yuan per year (i.e., ∼$1,500 – $20,000). Note that due to concerns over privacy, three participants did not report their parents’ annual income; these participants were, however, willing to report their parents’ highest level of education (*n* = 33). Objective SES based on parents’ salary was positively correlated with Objective SES based on parents’ highest attained level of education, *r*(31) = 0.450, *p* = 0.012. Past research on Objective SES recommends the use of parents’ highest attained level of education over parents’ annual income as an index of student Objective SES (Rosenberg, 1965), given that, in comparison to salary levels, education levels tend to be better predictors of other social factors such as self-esteem (Twenge and Campbell, 2002). As a result, we used parents’ highest attained level of education as our index of participants’ Objective SES below.

### Objective SES and TG Behavior

**FIGURE 5:**
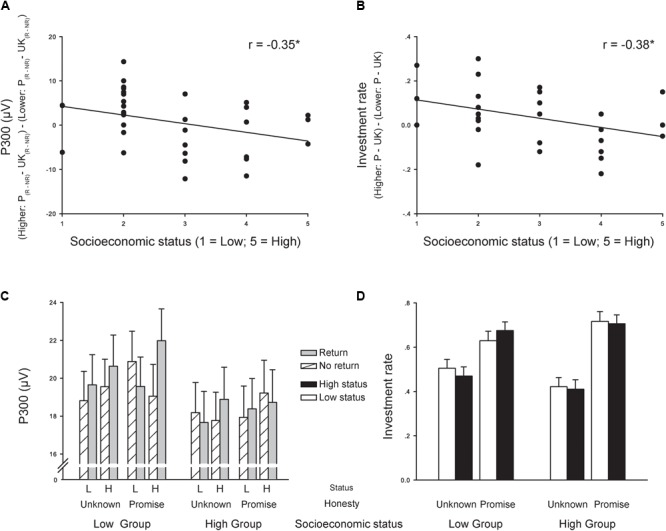
Effects of Objective SES (i.e., parents’ highest achieved level of education) on TG behavior and ERP response (P300 peak amplitudes in the medial central cluster time-locked to the TG feedback). **(A)** Correlation between Objective SES and the interaction between partner social status, promise, and return on P300 peak amplitudes. **(B)** Correlation between Objective SES and the interaction between partner social status and promise on investment behavior in TG. **(C)** Objective SES split-group analysis of P300 peak amplitudes plotted as a function of partner social status, promise, and return. **(D)** Objective SES split-group analysis of investment behavior plotted as a function of partner social status and promise. Groups based on median split: Low Objective SES group (*n* = 17) and High Objective SES group (*n* = 16). “P” indicates Promise; “UK” indicates Unknown; “L” indicates Lower Status Trustee; “H” indicates Higher Status Trustee.

There was a negative correlation between Objective SES and the TG behavior interaction between partner social status and promise conditions [i.e., (Higher status “promise” – Higher status “unknown”) – (Lower status “promise” – Lower status “unknown”)], *r*(31) = -0.381, *p* = 0.029 (**Figure 5B**). To better understand the effect of Objective SES on behavior, we used a median split (median = 2) to divide Objective SES into two groups: low Objective SES [education level of 1 and 2 (middle school – high school diploma), *n* = 17] and high Objective SES [education level of 3, 4, and 5 (trade school – graduate degree), *n* = 16]. We then entered Objective SES group (low vs. high) as a between-subjects factor along with two within-subjects factors [partner social status (lower vs. higher) and promise condition (“unknown” vs. “promise)] into a repeated-measures ANOVA. Adding Objective SES group as a between-subjects factor did not change the pattern of results described above (see *Behavioral Results*). There was a significant main effect of promise condition, *F*(1,31) = 59.132, *p* < 0.001, $\eta_{p}^{2}$ = 0.656, with participants investing more in the “promise” condition (0.682 ± 0.027) than in the “unknown” condition (0.452 ± 0.026). There was a non-significant trend or tendency for an interaction between partner social status and promise condition, *F*(1,31) = 3.863, *p* = 0.058, $\eta_{p}^{2}$ = 0.111. The pattern was the same as the pattern described above (see *Behavioral Results*). The interaction between Objective SES group and promise condition was significant, *F*(1,31) = 4.784, *p* < 0.036, $\eta_{p}^{2}$ = 0.134. Further tests showed that in the low Objective SES group, the difference between investment in the “unknown” condition (0.487 ± 0.042) and the “promise” condition (0.652 ± 0.043) was smaller ($\eta_{p}^{2}$ = 0.562), than in the high Objective SES group (“unknown” = 0.416 ± 0.031, “promise” condition = 0.711 ± 0.032, $\eta_{p}^{2}$ = 0.716).

Importantly, and in line with the negative correlation between Objective SES and the TG behavior interaction between partner social status and promise conditions, there was a non-significant trend or tendency for an interaction between Objective SES group, partner social status, and promise condition, *F*(1,31) = 3.667, *p* = 0.065, $\eta_{p}^{2}$ = 0.106 (**Figure 5D**). Further tests showed that the interaction between partner social status and promise condition was only significant in the low Objective SES group, *F*(1,16) = 8.302, *p* = 0.011, $\eta_{p}^{2}$ = 0.342, such that in the “promise” condition, low Objective SES participants invested more in higher status (0.675 ± 0.041) than lower status partners (0.629 ± 0.048), *p* = 0.045; in the “unknown” condition, there was no significant difference between investment in higher status (0.470 ± 0.045) and lower status partners (0.505 ± 0.045), *p* = 0.307. The interaction between partner social status and honesty condition was not significant in the high Objective SES group, *F* < 1, *p* = 0.973.

### Objective SES and MFN in the 250–310 ms Time Window Following TG Feedback

There was no correlation between Objective SES and the interaction between partner social status, promise condition, and return on the MFN in the 250–310 ms time window following TG feedback, *p* = 0.208. We do not report further analysis on the effects of Objective SES on MFN.

### Objective SES and P300 Peak Amplitudes Time-Locked to the TG Feedback

There was a negative correlation between Objective SES and the interaction between partner social status, promise condition, and return on the P300 peak amplitudes in the medial central cluster time-locked to the TG feedback, *r*(31) = -0.345, *p* = 0.049 (**Figure 5A**). We chose the medial central cluster because the P300 responses were largest on these electrodes. There is also a negative correlation if we test the correlation between Objective SES and the interaction between partner social status, promise condition, and return of the P300 peak amplitudes on the CPz electrode, *r*(31) = -0.358, *p* = 0.041. Similar to the analysis of Objective SES and TG behavior, to better understand the effect of Objective SES on average P300 peak amplitudes in the medial central cluster, we conducted a median split of Objective SES and entered Objective SES group (low vs. high) as a between-subjects factor along with three within-subjects factors [partner social status (lower vs. higher) and promise condition (“unknown” vs. “promise) and return (return vs. no return)] into a repeated-measures ANOVA. The pattern of results with Objective SES included as a between-participants factor are the same as those described above (see *P300 Following TG Feedback*). There was a significant main effect of partner social status, *F*(1,31) = 5.621, *p* = 0.024, $\eta_{p}^{2}$ = 0.153, with higher P300 amplitudes for higher status partners (19.475 ± 1.095) than for lower status partners (18.882 ± 1.070). There was also a significant interaction between partner social status and return *F*(1,31) = 7.284, *p* = 0.011, $\eta_{p}^{2}$ = 0.190. The pattern was the same as the pattern described above (see *P300 Following TG Feedback*).

There was a significant interaction between partner social status, promise condition, return, and Objective SES, *F*(1,31) = 11.086, *p* = 0.002, $\eta_{p}^{2}$ = 0.263 (**Figure 5C**). Further tests showed that the interaction between partner social status, promise condition, and return was only significant in the low Objective SES group, *F*(1,16) = 9.351, *p* = 0.008, $\eta_{p}^{2}$ = 0.369. In the high Objective SES group, the interaction between partner social status, promise condition, and return was not significant, *p* = 0.104. Further tests on the interaction in the low Objective SES group showed that in the “promise” condition, the interaction between partner social status and return was significant, *F*(1,16) = 13.050, *p* = 0.002, $\eta_{p}^{2}$ = 0.449: for higher status partners in the “promise” condition, P300 peak amplitudes were more positive-going in response to “return” feedback (21.984 ± 1.643) than “no return” feedback (19.049 ± 1.410), *p* = 0.001, whereas for lower status partners in the “promise” condition, there was no difference in P300 peak amplitudes in response to “return” feedback (19.559 ± 1.676) and “no return” feedback (20.881 ± 1.570), *p* = 0.188. In the “unknown” condition, the interaction between partner social status and return was not significant, *p* = 0.753. The pattern of the interaction between Objective SES group, partner social status, promise condition, and return is the same if we limit our analysis to ANOVA on the P300 peak amplitudes on the CPz electrode, *F*(1,31) = 11.836, *p* = 0.002, $\eta_{p}^{2}$ = 0.276.

## Discussion

In the current study, we used a modified version of TG to investigate whether and how social status influences evaluation of honesty-related feedback. At the behavioral level, participants tended to be more affected by promises given by higher status Trustees than lower status Trustees, despite receiving equal feedback about lower and higher status honesty. At the neural level, when viewing TG partner feedback, MFN in the time window of 250-310 ms was more negative-going when TG partners did not return than when they did return. P300 peak amplitudes differentiated higher status return feedback (i.e., return vs. no return), but did not do so for lower status partner return feedback; moreover, P300 responses were more positive-going for higher status partner returns than for lower status partner returns. Finally, participants in low Objective SES evidenced a greater P300 effect for higher status honesty than for lower status honesty and evidenced a tendency for investing more in promises given by higher status Trustees than lower status Trustees; neither of these effects were found in participants in high Objective SES. Taken together, these findings demonstrate that social status can modulate both the behavioral responses to and the neural processing of honesty-related feedback, and suggest that higher status honesty may be perceived as more motivationally salient or rewarding than lower status honesty in individuals with low Objective SES.

### Behavior

Despite the fact that participants viewed identical feedback across conditions (i.e., 50% return; 50% no return), participants invested substantially more in partners when promises were made to return at least half of the multiplied sum than when partners were not given the option to make a promise. Moreover, participants tended to be more affected by promises given by higher status than low status partners in TG. In particular, both lower and higher status promises increased the amount the participants invested in TG, in comparison with trials where promises were not available; however, higher status promises tended to increase investment to a greater extent. These behavioral findings provide support for the “social value” hypothesis, which predicts that participants would be more affected by promises given by higher status Trustees than by lower status Trustees. In contrast, the “expectation violation” hypothesis predicts that participants would be more surprised by higher status than lower status dishonesty and would thus invest less in higher status promises than lower status promises over time. No support was found for this hypothesis, and there was no evidence showing that participants’ investment behavior changed over time.

While the behavioral pattern found above suggests that higher status increases the influence of promises on investment behavior, this effect is relatively weak. Similar research measuring participants’ investment behavior in Trustees of lower and higher status in iterated one-shot TG found that participants invest significantly more in promises given by higher status Trustees than in promises given by lower status Trustees (Blue et al., under review). We suspect that the weakness of this effect in the current study was due to the feedback concerning the Trustee’s return behavior. In Blue et al. (under review), no feedback was given concerning whether the Trustee actually kept the promise, whereas here the participants roughly knew that the Trustee broke the promise in about half of the trials. Thus, the surprising finding was that even in such harsh conditions encouraging distrust, the participants still tended to trust the high status Trustee more than the low status Trustee, a pattern replicating Blue et al. (under review).

### MFN Effects on Outcome Feedback Evaluation

MFN responses were sensitive to outcome valence, as MFN responses were more negative-going for “no return” than for “return” feedback. This effect reinforces the notion that MFN encodes social expectancy violation, as not returning part of the multiplied sum is a violation of the trustworthiness norm. This trustworthiness norm in TG refers to the Investors’ tendency to send around 50% of the possible amount of money to Trustees (Berg et al., 1995; Johnson and Mislin, 2011), despite the unique Nash equilibrium prediction that the Investor, as a rational and self-interested agent, should transfer no money to the Trustee, given that a rational Investor should assume that the Trustee would act in a completely self-interested way (i.e., return none of the multiplied sum to the Investor). Thus, a large portion of Investors in TG expect Trustees to reciprocate their trusting behavior by acting in a trustworthy manner, and not returning may be interpreted as a violation of the trustworthiness expectation.

Interestingly, partner social status and the promise condition did not interact to influence MFN responses to outcome feedback, especially given that previous research shows that promise information modulates responses to TG outcome feedback (Ma et al., 2015). This may have been due to the forced feedback nature of the current experiment, as participants in Ma et al. (2015) were given feedback only if they invested in the Trustee, whereas in the current study, participants were given forced feedback. Indeed, we did find that the investment decision modulated MFN responses: if the analysis of MFN in the “promise” condition is restricted to invest-only trials, the MFN effect is even more pronounced than when the analysis includes all feedback, regardless of the investment decision.

There was also a main effect of promise condition on MFN responses to TG outcome feedback, as MFN responses were more negative-going for feedback in the “unknown” condition than in the “promise” condition. This main effect is most likely due to differences in investment behavior in the two conditions. Participants invested less in the “unknown” condition (investment rate = 45%) than in the “promise” condition (investment rate = 68%). Given that past research shows that distrust decisions elicit greater MFN responses to outcome feedback, in comparison with trust decisions (Long et al., 2012), and that investment behavior modulated MFN responses to TG outcome feedback, it is most likely that this main effect is driven by the participants decreased investment in the “unknown” condition than in the “promise” condition.

Finally, “return” and “no return” decisions were more directly tied to financial payoffs than promise and partner social status information, which could make this information more salient in early MFN outcome evaluation processing. This is in line with past research showing that, in social contexts, MFN encodes stimuli that are most directly tied to financial payoff, whereas social factors are left for later processing (e.g., P300 or LPP; Leng and Zhou, 2010; Wu et al., 2011b). This could mean that the outcome evaluation system may defer to a later, more top–down stage of processing to appraise the honesty-related outcomes in the context of social status, which would suggest that the outcome evaluation in TG may be composed of earlier, semi-automatic processing which is coarse in nature and provides discrete evaluations of return feedback regardless of its relation to honesty or social status, and later top–down controlled processing of outcome evaluation, where factors such as honesty and social status can undergo higher level cognitive appraisal.

### P300 Effects in Feedback Evaluation

In contrast to MFN, P300 responses to TG feedback were sensitive to the interaction between social status and return decisions. In particular, P300 responses differentiated return and no return feedback only for higher status Trustees. Moreover, higher status return feedback elicited greater P300 amplitudes than lower status return feedback. Given that P300 activity reflects affective/motivational significance (Nieuwenhuis et al., 2005; Leng and Zhou, 2010) and/or distribution of attention resources (Gray et al., 2004; Linden, 2005), the findings from the current study could suggest that higher status returns were more motivationally salient to participants than were lower status returns. Higher and lower status return likelihood and amounts were identical, which means that the increased P300 response to “return” outcomes from higher status Trustees than lower status Trustees could reflect increased perceived value or relevance of higher status “return” feedback, especially given that processing social status information is directly tied to reward-related processing in both human (Ly et al., 2011) and non-human primates (Deaner et al., 2005). Indeed, previous research using TG shows that Trustee characteristics, such as personal closeness to the Investor (i.e., “social value;” Fareri et al., 2015), modulate neural responses to Trustee feedback in brain areas related to reward processing, such as ventral striatum. Ventral striatum activity is also greater in responses to outcomes that result in reward which is shared with friends than reward which is shared with strangers (Fareri et al., 2012), suggesting that outcome evaluation is susceptible to influence of social reward. Research simultaneously measuring fMRI and EEG show that, during the anticipation of monetary gain, ventral striatum and P300 activity are positively correlated, suggesting that these two neural responses may be involved in similar motivational processing of reward-related stimuli (Pfabigan et al., 2014). Additionally, both ventral striatal and P300 activity are impaired in patients diagnosed with schizophrenia, and these impairments have both been shown to be associated with deficits in reward processing (Juckel et al., 2006; Vignapiano et al., 2016). Taken together, the P300 findings from the current study suggest that social status influences the motivational significance and/or attentional resources devoted to TG outcome feedback and that this modulation may reflect differences in perceived value of lower and higher status “return” outcomes in TG.

It is interesting that we did not find an interaction between partner social status, return, and promise information on P300 responses to TG feedback. We suspect that this may be due to individual differences in participant SES (Ly et al., 2011). Indeed, only participants in low Objective SES showed the expected interaction between partner social status, return, and promise information on P300 responses to TG feedback. In these participants, social status modulation of P300 responses to TG feedback was restricted to the “promise” condition, such that P300 responses were greater for higher status “return” than “no return” outcomes, whereas P300 responses were less sensitive to lower status promise feedback. These findings could suggest that, for participants in low Objective SES, higher status partner honesty feedback may be perceived as more motivationally salient and/or elicit greater attention allocation than lower status partner honesty feedback.

One potential explanation for these findings is that lower status individuals have the most to gain from high status cooperation in a social hierarchy (Cummins, 1996), and keeping promises is considered a sign of cooperation. This would be in line with the “social value” account and would suggest that one possible explanation for the increased P300 response to lower status than higher status honesty in participants with low Objective SES is that these individuals may value higher status honesty more than lower status honesty. Additionally, participants with low Objective SES evidenced a tendency for investing more in higher status than lower status promises, despite the equal reinforcement schedule. This behavioral finding provides further support for the “social value” account, as participants in low Objective SES may have believed they had more to gain by investing in higher status than lower status promises. Taken together, the behavioral and neural findings for low Objective SES participants could suggest that these individuals perceive higher status promises and honesty as being more valuable than that of their lower status counterparts. Given that we did not manipulate feelings of SES and given the non-significant trend or tendency of the SES behavioral interaction in the current study, future research could directly address whether changes in SES feelings (e.g., Subjective SES) could replicate the effects found in the current study and could provide more support for a causal explanation of SES in the current study.

In contrast to participants in low Objective SES, participants in high Objective SES evidenced no effects of partner social status on P300 responses or behavior. P300 responses were greater in the low Objective SES group than in the high Objective SES group, regardless of the condition, which could suggest that high Objective SES may have been associated with decreased attention to others’ social information, in general. Past research shows that individuals in high SES are less attentive to others’ information than individuals in low SES (Muscatell et al., 2012; Dietze and Knowles, 2016) and that high status individuals are more selective in their attention allocation (Shepherd et al., 2006). Attention-based differences between low and high SES individuals may be driven by different cognitive tendencies. Low SES individuals tend to have contextualist cognitive tendencies, whereas high SES individuals tend to have more individualistic cognitive tendencies and increased concern for goals and reward related to the self (Kraus et al., 2012). High status individuals are less reliant on others to achieve their goals, whereas low status individuals are more likely to help high status than fellow low status others, as the former is more valuable for attaining resources and protection in the future (Trivers, 1971; de Waal, 1989; Silk, 1992; Cummins, 1996, 2006; Stevens et al., 2005). Taken together, our findings regarding the effects of individual differences in Objective SES are in line with previous research, and suggest that, while high Objective SES participants are less concerned with others’ social status and honesty behavior, low Objective SES participants are especially attuned to higher status others’ honesty, an effect which is tied to increased investment likelihood in higher status than lower status promises.

A few points are worth mentioning. The social status manipulation (i.e., math quiz ranking) could, in fact, have influenced the way the participant viewed the lower and higher-ranking players in ways other than the prestige-based social status referred to in this study. (1) For example, despite the fact that we did not manipulate SES, participants did perceive higher ranking participants as having higher Subjective SES than that of their lower ranking counterparts. While this difference in perceived Subjective SES did not correlate with investment differences for lower and higher status partners in TG (*p* = 0.969), future research should look to manipulate partner SES while controlling for prestige-based social status to more directly address the unique effects of perceived SES on perceived trust. (2) Another possible explanation for the effect of social status on investment behavior may have been that participants may have inferred that higher status partners were happier than lower status partners after achieving their ranking (Hu et al., 2014), which could have increased perceived warmth and trustworthiness (Fiske et al., 2007). Despite the plausibility of this possibility, in the current study, participants evidenced a non-significant trend or tendency for perceiving higher status partners as *less* benevolent than their lower status counterparts, which is in line with past research (Dunn et al., 2012; Lount and Pettit, 2012), but does not support this alternative account. Moreover, status differences in perceived benevolence did not predict the TG behavior interaction between partner social status and promise conditions, and so we do not discuss it further. (3) Finally, another possible explanation is that high status partners were perceived as having put in more effort to the experiment, which could have increased their perceived trustworthiness. While we cannot rule this possibility out, it is important to note that the design of the experiment (only permitting 10 s per math question) rules out large differences in perceived effort. Taken together, the current study appears to be the start of a broader inquiry regarding the effects of social status on perceived trustworthiness.

## Conclusion

To conclude, by manipulating prestige-based social status, this study found that participants acting as Investors in TG tended to be more affected by higher status Trustee promises than by lower status Trustee promises, despite the equal reinforcement schedule across conditions. At the neural level, in an early time window (250–310 ms), MFN responses were sensitive to return outcome, as MFN amplitudes were more negative when partners did not return than when they did return. This effect was not modulated by the Trustee’s social status. In later processing, P300 responses *were* modulated by social status and return. P300 amplitudes were only sensitive to return feedback from higher status partners, and failed to distinguish lower status partner return feedback; moreover, P300 responses were more positive for higher status returns than lower status returns, which suggests that higher status positive feedback may have been perceived as more motivationally significant or rewarding than lower status positive feedback. The current study also found that the lower the participants’ Objective SES, the greater their differential P300 effect for higher status over lower status honesty and the more they invested in higher status than lower status promises, suggesting that individual differences in SES affect the perceived motivational salience/reward effect of social status on honesty. Taken together, we find that social status influences the effect of promises on investment behavior in TG, and that brain responses to honesty-related feedback in social hierarchies may involve both an early MFN processing of trustworthiness outcome valence information and a later P300 cognitive appraisal process which takes into account both social status and honesty and its relation to reward.

## Author Contributions

PB, JH, and XZ designed the experiments and wrote the manuscript. PB and JH collected the data.

## Conflict of Interest Statement

The authors declare that the research was conducted in the absence of any commercial or financial relationships that could be construed as a potential conflict of interest.
